# Sinomenine reduces iNOS expression *via* inhibiting the T-bet IFN-γ pathway in experimental autoimmune encephalomyelitis in rats

**DOI:** 10.7555/JBR.26.20110114

**Published:** 2012-06-30

**Authors:** Bingjie Gu, Yanying Zeng, Cheng Yin, Huijiuan Wang, Xiaofan Yang, Song Wang, Xiaohui Ji

**Affiliations:** a Department of Immunology, Nanjing Medical University, Nanjing, Jiangsu 210029, China;; b Department of Neurology, the First Affiliated Hospital, Nanjing Medical University, Nanjing, Jiangsu 210029, China.

**Keywords:** sinomenine, experimental autoimmune encephalomyelitis, iNOS, T-bet, interferon-γ (IFN-γ)

## Abstract

Sinomenine is a bioactive alkaloid isolated from the Chinese medicinal plant *Sinomenium acutum*. It is widely used as an immunosuppressive drug for treating rheumatic and arthritic diseases. In our previous studies, we found that sinomenine reduced cellular infiltration within the spinal cord and alleviated experimental autoimmune encephalomyelitis (EAE) in rats. In this study, we further investigated the mechanisms of sinomenine treatment in EAE rats. In EAE rats, treatment with sinomenine exerted an anti-inducible NO synthase (anti-iNOS) effect, which is related to the reductions of Th1 cytokine interferon-γ (IFN-γ) and its transcription factor, T-bet, in spinal cords. Moreover, sinomenine treatment of splenocytes stimulated with anti-CD3 antibody and recombinant rat interleukin 12 reduced the expression of T-bet and IFN-γ in vitro and also reduced the capability of supernatants of splenocyte culture to induce iNOS expression by primary astrocytes. However, sinomenine had no direct inhibitory effect on iNOS produced by astrocytes cultured with IFN-γ and tumor necrosis factor α *in vitro*. In conclusion, the anti-iNOS effect of sinomenine on EAE is mediated *via* the suppression of T-bet /IFN-γ pathway.

## INTRODUCTION

Multiple sclerosis (MS) is a chronic, neurodegenerative disease that is associated with central nervous system (CNS) demyelination[1]. Although the etiology of MS remains unknown, it is generally viewed as an autoimmune disease of the CNS[2]. Because the clinicopathological features of experimental autoimmune encephalomyelitis (EAE) are similar to those of human MS, EAE is commonly used to study the pathogenetic mechanisms of MS and to investigate the efficacy of potential therapeutic agents for MS[3].

CD4^+^ T cells are sensitized by the protein components of the myelin sheath, such as myelin basic protein (MBP), myelin oligodendrocyte glycoprotein (MOG), a glycoprotein believed to be important in the process of myelinization of nerves in the CNS, and proteolipid protein (PLP), the major myelin protein from the CNS. These encephalitogenic T-cells then migrate to the CNS, interact with microglia, astrocytes and a subpopulation of dendritic cells, and initiate plaque formation. The specific mechanisms that cause damage to the CNS not only involve the secretion of cytokines by T cells, but also the activation of glial cells to secrete both proinflammatory cytokines and inflammatory mediators such as tumor necrosis factor-α (TNF-α), interleukin (IL)-1β, IL-6 and nitric oxide (NO)[4],[5]. Overall, this process eventually leads to the destruction of the myelin sheath, axonal loss and damage. Excess NO is produced by the inducible NO synthase (iNOS), which can be induced by proinflammatory cytokines such as IFN-γ and TNF-α, and produced by monocytes/macrophages, glial cells and Th1 lymphocytes[6]. Expression of iNOS within the CNS correlates with disease activity and severity in both actively induced and adoptively transferred EAE[7].

Despite the availability of several immunomodulatory and immunosuppressive drugs, an efficient and effective treatment for MS is still urgently needed[8]. The alkaloid sinomenine (7,8-didehydro-4-hydroxy-3,7-dimethoxy-17-methyl-9α,13α,14α-morphinan-6-one) is a bioactive component derived from the Chinese medicinal plant *Sinomenium acutum*. It has been utilized by Chinese doctors to treat inflammatory and arthritic diseases for over 1,000 years[9]. We previously reported that sinomenine shows beneficial effects both by modulating the pathogenesis of EAE in Lewis rats and by decreasing the production of proinflammatory cytokines and chemokines such as IFN-γ, TNF-α, macrophage inflammatory protein (MIP), monocyte chemoattractant protein (MCP) and regulated upon activation normal T cell expressed and secreted (RANTES)[10]. However, the effects of sinomenine on iNOS remain unclear. In this study, we investigated the effects of sinomenine on the production of iNOS in an active EAE rat model. We found that sinomenine inhibited the T-bet/IFN-γ/iNOS axis in the spinal cord *in vivo*. In primary astrocytes, the anti-iNOS effect of sinomenine is correlated with blocking of IFN-γ, an important inducer of iNOS, which resulted from the inhibition of the transcription factor T-bet rather than direct suppression of iNOS production by astrocytes.

## MATERIALS AND METHODS

### Animals and reagents

Female Lewis rats (Vital River, Beijing, China) were maintained in a specific pathogen-free animal facility at the Laboratory Animal Center of Nanjing Medical University. All animal procedures were conducted in complete compliance with the NIH Guide for the Care and Use of Laboratory Animals and approved by the Nanjing Medical University. Sinomenine, of 98% purity verified by HPLC, was purchased from the National Institute of Pharmaceutical and Biological Products (Beijing, China). Recombinant rat IL-12, IFN-γ and TNF-α were purchased from Peprotech (Rocky Hill, NJ, USA). Anti-rat CD3 antibody was purchased from eBioscience (San Diego, CA, USA). Anti-T-bet antibody was purchased from Santa Cruz Biotechnology (Santa Cruz, CA, USA), and anti-iNOS antibody was purchased from Abcam (Cambridge, MA, USA).

### Induction of EAE and administration of sinomenine

Female Lewis rats were used at 6-8 weeks of age. EAE was induced in rats with MBP fragment 68-82 (MBP68-82; Sigma-Aldrich, St. Louis, MO, USA) as previously reported[10]. Rats were treated with either 0.2% dimethyl sulfoxide (DMSO), or sinomenine (50, 100 or 200 mg/kg/d) *via* intraperitoneal injection (i.p.) from d 1 to 3 for 5 consecutive d after immunization. Control rats (*n* = 6) were injected with incomplete Freund's adjuvant (IFA) supplemented with *Mycobacterium tuberculosis* emulsified with 0.2% DMSO plus pertussis toxin according to the same schedule.

### Determination of IFN-γ protein levels

The determination of IFN-γ concentrations in the spinal cords of rats and supernatants of splenocyte culture was performed using an ELISA kit (eBiosciences, San Diego, CA, USA) according to the manufacturer's instructions.

### Astrocyte culture and iNOS induction

Primary astrocyte-enriched cultures were prepared from the whole cortex of 1-day old Sprague Dawley rats. Briefly, the cerebral cortices were dissociated in Dulbecco's modified Eagle's medium (DMEM) containing 0.25% trypsin/EDTA (Invitrogen, Carlsbad, CA, USA), and then passed through a 70 µm pore nylon mesh (BD Biosciences, San Diego, CA). After centrifugation, the cell pellet was resuspended in DMEM/F12 containing 10% heat-inactivated fetal bovine serum (FBS; HyClone Laboratories Inc, Logan, UT), penicillin (50 U/mL), and streptomycin (50 µg/mL, Invitrogen). The cells (1×10^7^ cells/flask) were then placed onto poly-D-lysine-coated 75 cm^2^ tissue culture flasks. The medium was renewed every 2-3 d. Eight d later, the cells were shaken for 4 h on an orbital shaker to remove the microglia and then seeded onto multi-well tissue culture dishes. The cells were incubated with serum-free DMEM/F12 for 24 h before incubation with drugs. Cells were incubated with IFN-γ (2.5, 5 or 10 ng/mL, respectively) and TNF-α (2.5, 5 or 10 ng/mL, respectively) to induce the expression of iNOS. Additionally, the supernatant from splenocytes stimulated with anti-CD3 antibody and IL-12 in the presence (super1) or absence (super2) of sinomenine (1 mmol/L) added to the astrocytes to induce iNOS expression. Cells were analyzed for *iNOS* mRNA (for 6 h) by reverse transcription-PCR (RT-PCR) and protein (for 12 h) by Western blotting assays.

### Splenocyte culture and T-bet induction

Naïve splenocytes were isolated from Sprague Dawley rats and cultured at 37°C in a humidified atmosphere with 5% CO_2_ in RPMI 1640 (Sigma, Munich, Germany) supplemented with 10% (*V/V*) FCS, 2 mmol/L L-glutamine, 100 U/mL penicillin, and 100 µg/mL streptomycin (all from Sigma). For induction of T-bet, 2×10^6^ cells/well were stimulated with plate coated with anti-CD3 antibody (2 µg/mL) and recombinant rat IL-12 (10 ng/mL). To determine the effects of sinomenine, we pretreated cells with sinomenine (1 mmol/L) for 30 min. After 48 h, the supernatant was collected and assayed for IFN-γ production by ELISA. Cells were analyzed for *IFN-γ* and *T-bet* mRNA by RT-PCR (for 24 h) and T-bet protein by Western blotting assay (for 48 h).

### RT-PCR

Total RNA was isolated, and RT- PCR was employed to determine the mRNA level of *iNOS*, *IFN-γ* and *T*-*bet*. The PCR included a 5-min pre-incubation step at 95°C followed by amplification for 25 cycles at 94°C for 50 s, 59°C (for *IFN-γ*), 58°C (for *T*-*bet*), 52°C (for *iNOS*) for 50 s, 72°C for 1 min, and a final extension step at 72°C for 10 min. The sequences of primer pairs used to amplify rat *IFN-γ* (353 bp), *T*-*bet* (274 bp), *GAPDH* (421 bp) and *iNOS* (259 bp) were as follows: *IFN-γ* (forward 5′-TTTTGCAGCTCTGCCTCATG-3′ and reverse 5′-CTGTGGGTTGTTCACCTCGA-3′), *T*-*bet* (forward 5′-TCAGCTGAAAATCGACAACA-3′ and reverse 5′-CACTGCTCGGAACTCTGTTT-3′), *iNOS* (forward 5′-CTTTTAGAGACGCTTCTGAG-3′ and reverse 5′-TTTGATGCTTGTGACTCTTA-3′), *GAPDH* (forward 5′-ACTGCCACTCAGAAGACTGT-3′ and reverse 5′-TGCTGTAGCCATATTCATTG-3′). Values are presented as the relative amount of transcription of each sample normalized against the housekeeping gene.

### Western blotting assays

The proteins of rat spinal cords (100 µg) and cell extracts were run on 8% or 12% SDS-polyacrylamide gels, electro-transferred to a polyvinylidene difluoride (PVDF) filter, and blocked with 5% skimmed milk for 1.5 h. Rabbit anti-NOS2 polyclonal antibody or mouse anti-T-bet monoclonal antibody was used for primary blotting, horseradish peroxidase-conjugated anti-rabbit or anti-mouse IgG was used for secondary blotting. The proteins were detected by chemiluminescence using an ECL Western blotting detection kit according to the manufacturer's instructions. X-ray films (Kodak MXB Film) were exposed for 3 to 5 min. Quantification of the bands was carried out by densitometric analysis using Quantity One software (Bio-Rad, Hercules, CA, U.S.A.).

### Statistical analysis

The statistical analysis involving two groups was performed by means of Student's *t*-test, whereas analysis of variance (ANOVA) followed by Dunnett's multiple comparison test was used in order to compare more than two groups. All data were processed with SPSS software. The results were expressed as mean± S.D. *P* values less than 0.05 were considered statistically significant.

## RESULTS

### Sinomenine inhibits iNOS production in the spinal cords of EAE rats

**Fig. 1:**
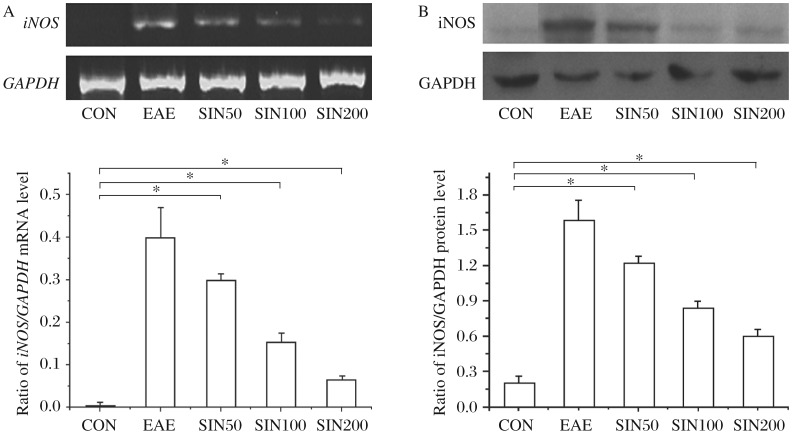
Sinomenine inhibits *iNOS* mRNA and protein expression in the spinal cord of EAE rats. Rats from the sinomenine-treated and control groups (*n* = 6 per group) were sacrificed on d 4 post onset. The mRNA and protein levels of iNOS in the spinal cord were quantified by RT-PCR (A) and immunoblotting (B). The results shown are mean±SD. for three independent experiments. A statistical evaluation was performed to compare the experimental groups and corresponding controls. *P < 0.05. CON: control group treated with incomplete Freund's adjuvant (IFA) supplemented with *Mycobacterium tuberculosis* emulsified with 0.2% DMSO plus pertussis toxin; experimental autoimmune encephalomyelitis (EAE) rats treated with 0.2% DMSO; SIN 50: EAE rats treated with 50 mg/kg sinomenine; SIN100: EAE rats treated with 100 mg/kg sinomenine; SIN200: EAE rats treated with 200 mg/kg sinomenine.

We examined the expression of iNOS in spinal cord sections from control, EAE, and sinomenine-treated EAE rats. As seen in ***Fig. 1***, MBP-induced EAE resulted in high expression levels of iNOS. Treatment of EAE rats with 50, 100 and 200 mg/kg sinomenine blocked the induction of iNOS at all doses tested.

### Sinomeninefails to inhibit iNOS production by primary astrocytes in vitro

**Fig. 2:**
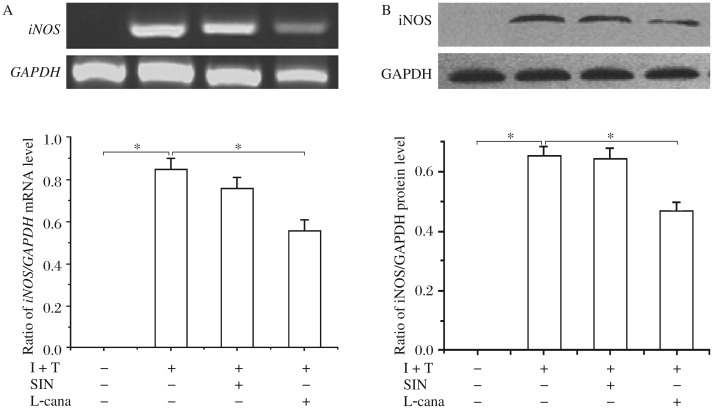
Sinomenine fails to directly inhibit iNOS production *in vitro* by primary astrocytes. Primary astrocytes were preincubated with sinomenine (1 mmol/L) for 30 min. Thereafter, cells were exposed to IFN-γ (10 ng/mL) and TNF-α (10 ng/mL) (I + T) either with or without sinomenine (1 mmol/L). Some cells were exposed to IFN-γ (10 ng/mL)/TNF-α (10 ng/mL) (I + T) and simultaneously to the selective iNOS inhibitor, *L*-canavanine (1 mmol/L). These cells were cultured for 6 h for RT-PCR (A) and 12 h for Western blotting (B). The results shown are mean±SD for three independent experiments. Significant differences between cells treated with TNF-α/IFN-γ alone and cells treated simultaneously with IFN-γ/TNF-α and sinomenine, or simultaneously with IFN-γ/TNF-α and *L*-canavanine, were analyzed. *P < 0.05. I+T: IFN-γ (10 ng/mL)+TNF-α (10 ng/mL); SIN: sinomenine; L-cana: *L*-canavanine.

Astrocyte cultures, pre-exposed (30 min) to sinomenine (1 mmol/L), were treated with a combination of IFN-γ and TNF-α. *L*-canavanine, an iNOS selective inhibitor, was used as a positive drug control. The *iNOS* mRNA transcript levels were greatly increased after exposure to 10 ng/mL IFN-γ and 10 ng/mL TNF-α, which was, however, attenuated by *L*-canavanine while sinomenine failed to abort IFN-γ and TNF-α-induced increase in *iNOS* mRNA transcript levels (***Fig. 2A***). Consistent with mRNA levels, iNOS protein levels were also significantly increased in astrocytes stimulated with IFN-γ and TNF-α; however, the levels of iNOS in the primary astrocytes cultures were reduced by *L*-canavanine, but not by SIN (***Fig. 2B***).

**Fig. 3:**
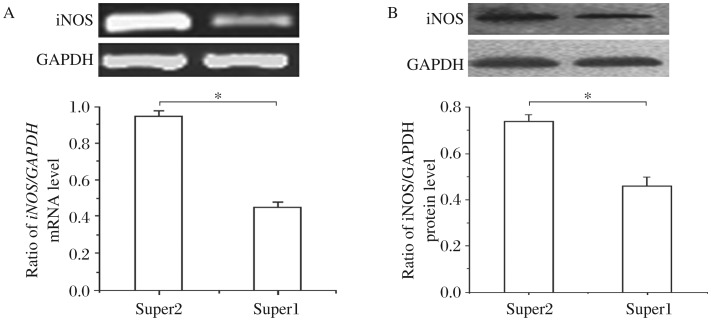
Supernatants of splenocytes cultured simultaneously with anti-CD3 antibody, IL-12 and sinomenine inhibit iNOS production by primary astrocytes. The supernatants of rat splenocytes stimulated with anti-CD3 antibody and IL-12 in the presence (super1) and absence (super2) of sinomenine (1 mmol/L) were collected and then used to induce iNOS production by primary astrocytes. A: RT-PCR was performed with the cellular RNA extracts to determine the expression levels of *iNOS* mRNA. B: Western blotting was performed with the cellular protein extracts to assay the level of iNOS protein. The results shown are mean±SD for three independent experiments. Significant difference between the effects of supernatants of splenocytes cultured with anti-CD3 antibody and IL-12 and simultaneously with anti-CD3 antibody, IL-12 and sinomenine on iNOS production by primary astrocytes was analyzed.*P < 0.05.

### Sinomenine suppresses anti-CD3 antibody/IL-12 induced iNOS production by primary astrocytes

According to the above results, sinomenine had no direct effect upon *iNOS* mRNA and protein levels, so we speculated that sinomenine may have an indirect inhibitory effect on iNOS production by primary astrocytes. To investigate this, we used supernatants from splenocytes stimulated with anti-CD3 antibody and IL-12 in the presence, or absence, of sinomenine (1 mmol/L) to mimic the interaction between astrocytes and the cytokines secreted by T-cells. Interestingly, the supernatants from the culture of splenocytes treated simultaneously with anti-CD3 antibody, IL-12 and sinomenine induced significantly lower production of iNOS by primary astrocytes compared with those treated only with anti-CD3 antibody and rmIL-12. (***Fig. 3A*** and ***3B***).

### Sinomenine inhibits the production of IFN-γ and T-bet in the spinal cords of EAE rats and primary splenocytes

**Fig. 4:**
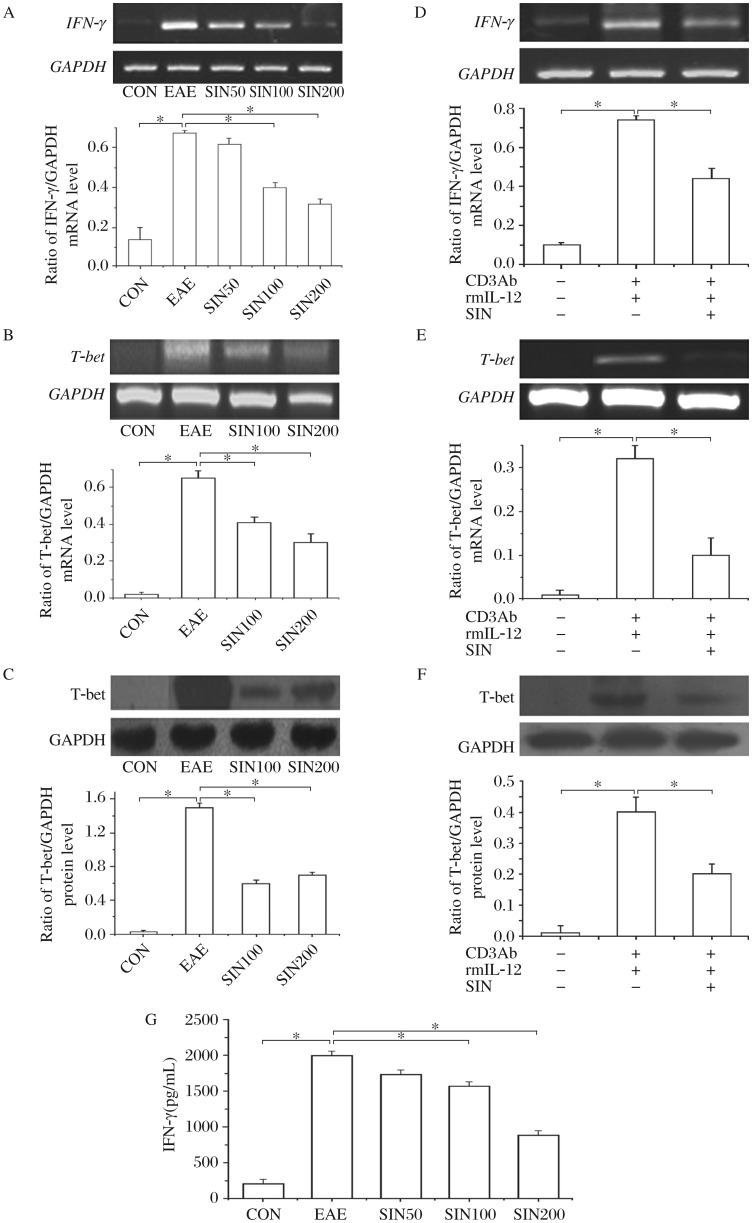
Sinomenine decreases the levels of IFN-γ and T-bet in the spinal cords of EAE rats and primary splenocytes. Cytokine levels in the spinal cords from EAE animals with and without sinomenine (SIN) treatment (*n* = 6 per group) were analyzed at d 4 post-onset. *IFN-γ* mRNA (A, product is 353 bp with 421 pb GAPD as internal control) and *T*-*bet* mRNA (B, product is 274 bp with 421 pb GAPD as internal control) levels were determined by RT-PCR, and T-bet protein levels (C) were quantified by Western blotting, and IFN-γ levels by ELISA (G). The results shown are mean±SD for three independent experiments. Primary splenocytes from Sprague Dawley (*n* = 6) were cultured with the stimulation of anti-CD3 antibody and IL-12, in the presence or absence of SIN (1 mmol/L). After 24 h, the cells were harvested and analyzed for *IFN-γ* (D, product is 353 bp with 421 pb GAPD as internal control) and *T*-*bet* mRNA (E, product is 274 bp with 421 pb GAPD as internal control). After 48 h, cells were harvested and analyzed for T-bet protein (F). *P < 0.05.

The *IFN-γ* mRNA levels in the spinal cords were increased in the EAE group, compared with the control group. Sinomenine at 100 and 200 mg/kg/d significantly downregulated *IFN-γ* mRNA levels (***Fig. 4A***). The IFN-γ protein level in the spinal cords of EAE rats was 2,000±302.0 pg/mL by ELISA, which was significantly increased when compared with that of control rats (213.3±60.6 pg/m *P* < 0.05,***Fig. 4G***). Treatment with sinomenine at 100 or 200 mg/kg/d reduced IFN-γ protein production by 21.32% or 55.29%, respectively (*P* < 0.05), and the concentration of IFN-γ was 1,573±218.5 pg/ml and 894.0±194.7 pg/mL (***Fig. 4G***). T-bet was not detected in the spinal cords of control rats, but was found at an appreciable level in EAE rats (***Fig. 4B*** and ***C***). Sinomenine, at 100 and 200 mg/kg/d, unequivocally suppressed the expression of T-bet in the spinal cords of EAE rats (***Fig. 4B*** and ***4C***).

To confirm whether sinomenine has had a direct immunomodulatory effect on T cells, we studied its effects on the expressions of IFN-γ and T-bet in primary splenocytes. The concentration of IFN-γ by ELISA in the supernatant of splenocytes stimulated with anti-CD3 antibody and rmIL-12 was 758.8±19.19 pg/mL, which was significantly higher than naïve splenocytes (64.57±19.1 pg/mL, *P* < 0.05). Treatment with sinomenine (1 mmol/L) significantly reduced IFN-γ level in the supernatant, which was 618.1±12.23 pg/mL compared with that in the stimulation group without sinomenine treatment (*P* < 0.05). As shown in Fig. 4, naïve splenocytes expressed *IFN-γ* mRNA (***Fig. 4D***), *T*-*bet* mRNA (***Fig. 4E***) and protein (***Fig. 4F***) at basal levels. However, stimulation with CD3 antibody under polarized conditions with IL-12 induced their expression significantly. Treatment with sinomenine (1 mmol/L) markedly inhibited the expression of both IFN-γ and T-bet by splenocytes.

## DISCUSSION

Sinomenine is an alkaloid extracted from the Chinese medicinal plant *sinomenium acutum*. Previous pharmacological studies have shown that sinomenine has significant anti-inflammatory, immunosuppressive, and anti-angiogenic properties[11],[12]. For example, sinomenine has been found to decrease both *TNF-α* and *IL*-*1*β mRNA expression by inhibiting NF-κB binding activity in rats with adjuvant arthritis[13]. In addition, sinomenine exerts anti-arthritic effects through suppression of both Th1 and Th2 immune responses, which is related to enhanced secretion of TGF-β[14]. We have also demonstrated that sinomenine inhibits the production of both IFN-γ and TNF-α within the CNS and in the splenocytes of EAE rats (10). Recently, sinomenine was found to cause a decrease in *T*-*bet* mRNA expression and to elicit a decrease in the serum levels of IFN-γ in mesangial proliferative nephritis patients[15]. However, little is known about the effects of sinomenine on iNOS expression in EAE, and the interaction between sinomenine and astrocytes.

MS is an inflammatory, demyelinating, neurodegenerative disease of the CNS[16]. T cells specific for autoantigens such as MBP and MOG are activated in the periphery. Infiltration by lymphocytic and mononuclear cells into the CNS, breakdown in the blood-brain barrier permeability, astrocyte hypertrophy, and demyelination are important features of both MS and EAE[17]. Once inside the CNS, leukocytes act upon astrocytes. For example, autoreactive and activated T cells secrete IFN-γ and TNF-α, initiating the production of inflammatory mediators such as IL-1 and IL-6, and excess synthesis of (NO) by astrocytes. NO is catalyzed by NO synthase (NOS), which is divided into a constitutive form (eNOS or nNOS) and an inducible form (iNOS). IFN-γ has been shown to be the major inducer of iNOS. With respect to CNS cells, IFN-γ combined with TNF-α is sufficient to induce NO release from astrocytes[18]. IFN-γ, TNF-α, and excess NO are of central importance in the pathogenesis of both MS and EAE[19],[20],[21]. The expression of iNOS is increased in microglia and astrocytes in the CNS of EAE rats[22]. The pathogenetic role of iNOS in the development of EAE is supported by the fact that antisense knockdown of iNOS and inhibitors of iNOS ameliorate EAE[23].

In this study, we found that sinomenine reduced iNOS production in the spinal cords of EAE rats. Therefore, we investigated the effects of sinomenine on iNOS production by astrocytes *in vitro*. The astrocytes, stimulated with IFN-γ and TNF-α, induced iNOS production that was not inhibited by sinomenine. We suspected that sinomenine may indirectly inhibit the induction of iNOS by affecting the microenvironment of the astrocytes. We cultured astrocytes in the supernatants from splenocytes to mimic the microenvironment in the CNS, in which cytokines secreted by T cells act upon astrocytes and induce excess iNOS production. We found that astrocytes, incubated with supernatants from splenocytes cultured in the presence of anti-CD3 antibody combined with IL-12, significantly increased their production of iNOS, while those incubated under the same conditions, but also treated simultaneously with sinomenine, produced much lower levels of iNOS. In a previous study, we demonstrated that sinomenine inhibits MBP-induced IFN-γ and TNF-α production by splenocytes in EAE rats[10]. Taken together, these interesting phenomena suggest that the inhibitory effect of sinomenine on IFN-γ may contribute to the inhibition of iNOS expression by astrocytes in the CNS.

To further understand the mechanism of this negative regulation of CNS inflammation and the anti-iNOS effects of SIN, we examined its direct effect on T cell responses. We found that treatment with sinomenine *in vivo* decreased IFN-γ expression in the spinal cords of EAE rats. This result is consistent with other reports showing that sinomenine inhibits IFN-γ levels in MOG induced EAE mice[24]. T-bet, a Th1-specific T box transcription factor, controls the expression of Th1 cytokines and initiates Th1 lineage development from naive Th0 cells[25]. Previous reports suggest that T-bet plays a critical role in the progression of EAE, as it can promote Th1 development and IFN-γ production[26]. *In vitro* suppression of T-bet during myelin-specific T cell differentiation, and *in vivo* administration of T-bet-specific anti-sense oligonucleotides, or siRNA, ameliorate EAE[27]. *T*-*bet*-deficient mice immunized with MOG are resistant to the development of EAE, with minimal inflammatory infiltrates in the CNS. In this study, sinomenine curtailed the expression of T-bet in the spinal cords of EAE rats, suggesting that the inhibition of T-bet expression by sinomenine might contribute to the modulation of IFN-γ production. To determine the molecular mechanisms involved in the inhibition of primary T cell responses, we examined the effect of sinomenine on anti-CD3 antibody and IL-12 inducing T-cells activation and T-bet induction *in vitro*. We demonstrated that the inhibitory effects of sinomenine on activation and IFN-γ production of splenocytes were not due to the cytotoxicity of sinomenine, according to the fact that no cytotoxicity of splenocytes was found in treatment with sinomenine at 1 mmol/L, as indicated by the WST assay (data is not shown, so we chose the high concentration of 1 mmol/L to be consistent with other reports[28]). Sinomenine suppressed the production of IFN-γ mediated by anti-CD3 antibody and IL-12, and this suppression was associated with the inhibition of the Th1 transcription factor, T-bet.

These results suggest that sinomenine inhibits T cell activation and alters the cytokine profile in the CNS microenvironment. Sinomenine inhibited the production of iNOS through T-bet/IFN-γ suppression. Thus, sinomenine might be a potential agent for MS and other inflammatory autoimmune diseases.

## References

[1] Buck D, Hemmer B (2011). Treatment of multiple sclerosis: current concepts and future perspectives. J Neurol.

[2] Sospedra M, Martin R (2005). Immunology of multiple sclerosis. Annu Rev Immunol.

[3] Gold R, Linington C, Lassmann H (2006). Understanding pathogenesis and therapy of multiple sclerosis via animal models: 70 years of merits and culprits in experimental autoimmune encephalomyelitis research. Brain.

[4] Frischer JM, Bramow S, Dal-Bianco A, Lucchinetti CF, Rauschka H, Schmidbauer M (2009). The relation between inflammation and neurodegeneration in multiple sclerosis brains. Brain.

[5] Dutta R, Trapp BD (2007). Pathogenesis of axonal and neuronal damage in multiple sclerosis. Neurology.

[6] Saha RN, Pahan K (2006). Signals for the induction of nitric oxide synthase in astrocytes. Neurochem Int.

[7] Teixeira SA, Castro GM, Papes F, Martins ML, Rogerio F, Langone F (2002). Expression and activity of nitric oxide synthase isoforms in rat brain during the development of experimental allergic encephalomyelitis. Brain Res Mol Brain Res.

[8] Bosnjak-Pasic M, Vidrih B, Miskov S, Demarin V (2009). Treatment of multiple sclerosis. Acta Clin Croat.

[9] Liu L, Buchner E, Beitze D, Schmidt-Weber CB, Kaever V, Emmrich F (1996). Amelioration of rat experimental arthritides by treatment with the alkaloid sinomenine. Int J Immunopharmacol.

[10] Zeng Y, Gu B, Ji X, Ding X, Song C, Wu F (2007). Sinomenine, an antirheumatic alkaloid, ameliorates clinical signs of disease in the Lewis rat model of acute experimental autoimmune encephalolmyelitis. Biol Pharm Bull.

[11] He X, Wang J, Guo Z, Liu Q, Chen T, Wang X (2005). Requirement for ERK activation in sinomenine-induced apoptosis of macrophages. Immunol Lett.

[12] Kok TW, Yue PY, Mak NK, Fan TP, Liu L, Wong RN (2005). The anti-angiogenic effect of sinomenine. Angiogenesis.

[13] Wang Y, Fang Y, Huang W, Zhou X, Wang M, Zhong B (2005). Effect of sinomenine on cytokine expression of macrophages and synoviocytes in adjuvant arthritis rats. J Ethnopharmacol.

[14] Feng H, Yamaki K, Takano H, Inoue K, Yanagisawa R, Yoshino S (2007). Effect of sinomenine on collagen-induced arthritis in mice. Autoimmunity.

[15] Cheng Y, Zhang J, Hou W, Wang D, Li F, Zhang Y (2009). Immunoregulatory effects of sinomenine on the T-bet/GATA-3 ratio and Th1/Th2 cytokine balance in the treatment of mesangial proliferative nephritis. Int Immunopharmacol.

[16] Hemmer B, Nessler S, Zhou D, Kieseier B, Hartung HP (2006). Immunopathogenesis and immunotherapy of multiple sclerosis. Nat Clin Pract Neurol.

[17] Berger C, Hiestand P, Kindler-Baumann D, Rudin M, Rausch M (2006). Analysis of lesion development during acute inflammation and remission in a rat model of experimental autoimmune encephalomyelitis by visualization of macrophage infiltration, demyelination and blood-brain barrier damage. NMR Biomed.

[18] Lowenstein CJ, Padalko E (2004). iNOS (NOS_2_) at a glance. J Cell Sci.

[19] Petermann F, Korn T (2011). Cytokines and effector T cell subsets causing autoimmune CNS disease. FEBS Lett.

[20] Xiao BG, Ma CG, Xu LY, Link H, Lu CZ (2008). IL-12/IFN-gamma/NO axis plays critical role in development of Th1-mediated experimental autoimmune encephalomyelitis. Mol Immunol.

[21] Pozza M, Bettelli C, Aloe L, Giardino L, Calza L (2000). Further evidence for a role of nitric oxide in experimental allergic encephalomyelitis: aminoguanidine treatment modifies its clinical evolution. Brain Res.

[22] Smith KJ, Lassmann H (2002). The role of nitric oxide in multiple sclerosis. Lancet Neurol.

[23] Dalton DK, Wittmer S (2005). Nitric-oxide-dependent and independent mechanisms of protection from CNS inflammation during Th1-mediated autoimmunity: evidence from EAE in iNOS KO mice. J Neuroimmunol.

[24] Yan LC, Bi EG, Lou YT, Wu XD, Liu ZD, Zhou J (2010). Novel sinomenine derivative 1032 improves immune suppression in experimental autoimmune encephalomyelitis. Biochem Biophys Res Commun.

[25] Szabo SJ, Kim ST, Costa GL, Zhang X, Fathman CG, Glimcher LH (2000). A novel transcription factor, T-bet, directs Th1 lineage commitment. Cell.

[26] Yang Y, Weiner J, Liu Y, Smith AJ, Huss DJ, Winger R (2009). T-bet is essential for encephalitogenicity of both Th1 and Th17 cells. J Exp Med.

[27] Lovett-Racke AE, Rocchini AE, Choy J, Northrop SC, Hussain RZ, Ratts RB (2004). Silencing T-bet defines a critical role in the differentiation of autoreactive T lymphocytes. Immunity.

[28] Shu L, Yin W, Zhang J, Tang B, Kang YX, Ding F (2007). Sinomenine inhibits primary CD4+ T-cell proliferation via apoptosis. Cell Biol Int.

